# Perceptions of parental involvement in youth handball players, the effects of sport participation stage and sports injury

**DOI:** 10.3389/fpsyg.2024.1412116

**Published:** 2024-06-03

**Authors:** Krisztina Kovács, Johanna Takács, István Juhász, Katalin Kovács

**Affiliations:** ^1^Department of Psychology and Sport Psychology, Institute of Economic and Social Sciences, Hungarian University of Sports Science, Budapest, Hungary; ^2^Department of Social Sciences, Faculty of Health Sciences, Semmelweis University, Budapest, Hungary; ^3^Hungarian Handball Federation, Budapest, Hungary; ^4^Faculty of Education and Psychology, Institute of Health Promotion and Sport Sciences, Eötvös Lóránd University, Budapest, Hungary

**Keywords:** parental involvement, sports injury, sport participation stage, family characteristics, Youth athletes

## Abstract

**Introduction:**

Globally, from the age of 14, the dropout rate of young athletes is high in all sports games in Hungary. The reasons for dropping out are complex, however parental support is critical to succeed or continue, especially after failure or injury. The present study explored the main effects of sports injury and sport participation stage on parental involvement in sports.

**Methods:**

1,174 parents and 690 athletes completed our questionnaire, which contains questions on young players’ sport participation, injury background and Parental Involvement in Sport Questionnaire.

**Results:**

Parents’ self-perceived level of involvement differed from the parental involvement perceived by their children. The significant predictors were the person who completed, the parent/athlete, the athlete’s previous sports injury and the child’s current stage of sport participation. In Directive Behavior, the main effect of stages is only seen in parents whose child has been injured. In the sample of injured athletes, the rate of perceived parental Praise and Understanding tends to be lower in the specializing stage.

**Discussion:**

Our findings suggest that these two behaviors could be part of the same parenting style, which requires further investigation. The results expand the existing knowledge of the complexity of parents’ involvement in children’s sports careers. These findings have implications beyond parental psychoeducation impacting the work of coaches, sports physicians and rehabilitation experts.

## Introduction

Parents play a fundamental role in supporting their children’s sports experiences and careers (Knight and Holt, 2014) and helping them be involved and committed (Holt and Knight, 2014; Dunn et al., 2016). Research shows that similar to how young athletes are affected by their parents’ attitudes and actions towards sports, parental behavior can also be influenced by their children’s sports experiences (Lally and Kerr, 2008; Brown et al., 2019). The impact of parental involvement in their child’s sporting life is one of the most common themes in sports parent studies. One of the primary focuses in early sports parent research, was the characterization of parental behavior, whereas more recent studies have concentrated on comprehending the characteristics and factors that underlie various parental behaviors and how they interact with their child’s participation in sports (Dorsch et al., 2021). Through parents’ sport socialization, parents change their behavior, affect and cognition as a result of the organized sport participation of their children (Dorsch et al., 2009). Parental involvement is an umbrella term covering a variety of behavioral, cognitive and affective components of parenting that directly or indirectly influence children’s development and performance (Grolnick and Slowiaczek, 1994). It is a multidimensional construct including parental beliefs and feelings about the child’s activity, which are manifested in active support for the child or in pressure to meet parental expectations (Leff and Hoyle, 1995).

Lee and Maclean (1997) defined four subtypes of parental involvement by the intensity and quality of characteristic parental behaviors including directive behavior (DB), parental pressure (PP), active involvement (AI), and praise and understanding (PU). These four subtypes comprise two main types of parental involvement. DB and PP are the two aspects of parental pressure as a main type. The former is aimed at directly controlling the child’s sport-related behavior, while the latter is based on expectations for good performance and/or success in competition. PU and AI are the two components of parental support. PU includes empathetic and understanding parental behaviors, while AI is manifested in parental attendance at the child’s training sessions and competitions. Most studies have examined parental involvement in sports from the children’s perspective. Only a few previous studies have addressed both the parents’ and children’s perspectives on the characteristics of parental involvement examining a relatively small sample of athletes/parents (Marsh et al., 2015; Goodman and James, 2017).Studies have shown that supportive parenting behaviors were the significant predictor on adolescent athletes, such as commitment and sports values (Danioni et al., 2017), while PP can indicate a positive relationship with higher levels of state anxiety (Bois et al., 2009; O’Rourke et al., 2011) and reduced feelings of enjoyment (Stein et al., 1999; Henriksen et al., 2010). The psychological impact of DB depends on both the child’s age and the situation: it can help young athletes overcome difficulties or cause them to feel anxious (Giannitsopoulou et al., 2010).

Côté and Hay (2002) offer a developmental model of sport participation which consists of three active phases (sampling—between 8–12 years; specializing—between 12–16 years and investment—over 16 years). Studying the conditions for a successful career transition, Wuerth et al. (2004) found a need for more intense DB (from the sampling to the specializing stage, between ages 12–14) stage, while Giannitsopoulou et al. (2010) found an increased need PU at the specializing stage. Researchers have shown that perceived parental involvement was associated with athletes’ sport participation stages (Côté and Hay, 2002; Wylleman and Lavallee, 2004), and parental involvement decreases with the child’s progress in sports. The risk of sports injury emerges as a significant parental stressor and their children’s previous injuries might influence parental involvement (Podlog et al., 2023). Discrepancies in perspective have also been observed for variables that include the level of PU and DB, relationship warmth and conflict, and parent positive and negative affect (Wuerth et al., 2004; Dorsch et al., 2016).

Research has also been conducted to examine the characteristics of parental involvement in terms of wider socio-contextual characteristics as well as individual factors. Individual factors include parental sports background (Dorsch et al., 2009; Knight et al., 2016), family income (Baxter-Jones et al., 2003), educational background and marital status (Kovács et al., 2022), while environmental factors include the characteristics of the sport and sports injury (Dorsch et al., 2009; Harwood and Knight, 2016; Knight et al., 2016). Dorsch et al. (2009) revealed six moderators of parental involvement including the child’s age, the parent’s previous sporting experience, the child’s and the parent’s gender, the child’s temperament and personality, the intensity of support received from the immediate environment, and the type of sport pursued (individual vs. team sport). The direct impact of parental involvement was found to decrease with the child’s age, while the influence of peers and coaches became an increasingly important social force (Knight and Holt, 2012).

### Study objectives

In our study, we examined what factors might predict changes in parental involvement from both the parents’ and children’s perspectives. According to the Bioecological Model of Human Development proposed by Bronfenbrenner (1995) and Bronfenbrenner and Morris (1998) parents are part of their child’s microsystem, that is, they have a direct and indirect impact on their child’s sports career, while their parental attitudes and behavior may be substantially influenced by other systems (e.g., previous sport experiences, expectations at the workplace, or their relationship with the child’s sports club). Most research separately analyses the parent’s perspective and the child’s perceived experience. Thus in the present study, the parents’ and athletes’ responses were analyzed separately to highlight differences in their perceived experiences and to examine the parental involvement as well as the effect of sports injury and sport participation stage. Since parental involvement can be influenced by the type of sport (Dorsch et al., 2009), we conducted our research on a specific sport, handball. The number of training sessions and the quality of the training may also be determinants of parental involvement (Padaki et al., 2017). In Hungary, academic training (typically including boarding school education) is separated from club training. Therefore, we excluded academic handball training sessions and focused only on club-based training sessions. In sum, the present study aimed to explore the parental involvement of both athletes’ and parents’ perspectives concurrently.

## Materials and methods

### Procedure

The survey was carried out by convenience sampling. For parents, the following inclusion criteria were included (a) having at least one child who played handball (b) their children aged between 12 and 18 years of age; (c) their children were registered members of a sports club at the time of data collection. Exclusion criterion: the child was registered with a handball academic team. For youth athletes, inclusion criteria included (a) the type of sport was handball (b) aged between 12 and 18 years of age; (c) being a registered member of a sports club at the time of data collection. The exclusion criteria were registered as a handball academic team. Initial contact was made with the representatives of youth teams/clubs to obtain their permission to approach athletes regarding the study. Researchers contacted parents through clubs associated with the Hungarian Handball Federation. The research was conducted with an online questionnaire system, the filling out lasted approximately 10–15 min. There were different online platforms for the athletes’ and for the parents’ questionnaires. First, participants were informed about the goal and the content of the study and ethical standards such as confidentiality, then they were asked to check a box if they agreed to continue and participate. They were assured about their anonymity. Participants were informed that enrolling in this study is completely voluntary and that they may stop participation at any time. The first part of the questionnaire contained questions regarding demographic and sport-related data. In the last part, the items of the PISQ were presented. The research was conducted in accordance with the Declaration of Helsinki and approved by the Research Ethics Committee of the ELTE PPK; with license number 2022/321. Data were collected between June 2022 and July 2022.

### Participants

Parents and athletes (*N* = 1871) were recruited with the support of the Hungarian Handball Federation. All parents had at least one child who played handball regularly as a registered member of a sports club at the time of data collection. The study sample included 1864 parents and athletes, seven parents were excluded based on missing data. The parents (283 males and 898 females) were between 28 and 55 years old (M = 44, SD = 4.62). 55.4% of the parents were former competitive athletes themselves. The mean age of their children was 13.66 years (SD = 1.69, range 12–18 years). All athletes played handball regularly as registered members of a sports club at the time of data collection in the same competition level. At the time of data collection, 226 male and 464 female players were aged between 12 and 18 years (M = 14.44, SD = 1.78). The average number of years they played handball was 5.90 (SD = 2.69).

### Measurement instrument

The participants provided data on both their own and their children’s sociodemographic and sport-relevant characteristics (gender, age, and past sporting experiences; children’s gender, age, sport, sport participation stage as defined by Côté and Hay (2002), and past injuries). Inclusion criteria for injury were considered injuries in the previous competitive season that required a rehabilitation period of at least 2 months.

Parental Involvement in Sport Questionnaire (PISQ)—the Hungarian version of the Parental Involvement in Sport Questionnaire (PISQ; Lee and Maclean, 1997; Kovács et al., 2020) adapted for both parent and athlete samples. Both of the questionnaires consist of 14 items on four subscales: Directive Behavior (parental version: 6 items, athlete version 4 items; “Do your parents/you tell you/your child how they think you can improve your technique?”), Active Involvement (both parental and athlete version: 2 items; “Do your parents/you volunteer to help at competitions as officials, whips etc.?”), Praise and Understanding (parental version: 2 items, athlete version 5 items;” After a match do your parents/you praise you/your child for trying hard?”), Parental Pressure (parental version: 4 items, athlete version 3 items; “Do your parents/you put pressure on you/your child concerning your/his or her sport?”) subscales. Each Likert item is rated on a five-point scale. The Hungarian version of the PISQ showed acceptable–good internal consistency (parental version Cronbach’s α = 0.61 to 0.86; athlete version Cronbach’s α = 0.63 to 0.83). Since the subscales of the PISQ questionnaire for parents and athletes differ in terms of the number of items but are identical in the content of the constructs, the scores of the PISQ subscales of athletes and parents were divided by the number of items in subsequent analyses. A higher score indicates a higher level of the given parental involvement in their children’s sports careers.

### Statistical analysis

In the present study, we used *a priori* power analysis to calculate the sample size for a survey with a 0.95 confidence interval, 0.04 margin of error, and 0.95 confidence interval (Statistics Kingdom 2017^1^). The calculated sample size was 601. During sampling, the sample size was doubled considering missing values, invalid cases and outliers. Finally, based on the inclusion criteria, the required sample size was respected in the present study (*n* = 1,174 parents, after excluding cases with missing data and *n* = 690 athletes). Descriptive statistics and frequency data were used to describe the main factors, sport participation stage (Stages) and previous sports injury (Injury) in the samples of the parents and athletes (Samples). The assumptions, normality for each group of the independent variable, and homogeneity of variances were checked before running the statistical tests. These assumptions were met or robust tests were used. To examine the differences between parents and athletes on the four PISQ subscales, Welch’s independent samples t-tests with Bonferroni correction were conducted with Hedges’ g effect size measurement. The association between sport participation stage and the athletes’ previous sports injuries was tested by Pearson’s chi-square test with Cramer’s V measure of association for the strength of association. A factorial analysis of variance was conducted by calculating partial eta squared effect size measurement to explore the main effects of the sport participation stage and the athlete’s previous sports injury on PISQ. We examined the main effects of the Stages and Injury, the Stages × Injury 2-way interaction, and the Stages × Injury × Samples 3-way interaction. All statistical analyses were performed with IBM SPSS Statistics for Windows, Version 26.0 (IBM Corp. Released 2019. Armonk, NY).

## Results

### Sport participation stage and previous sports injury

Frequency distribution of sport participation stage (Stages) and previous sports injury (Injury) in the sample of parents and athletes, see Table 1.

**Table 1:** Frequency distribution of stages and injury in the samples of parents and athletes.

	Parents (*n* = 1,174)	Athletes (*n* = 690)
Stages, % (*n*)		
Sampling	55.2 (647)	34.9 (241)
Specializing	28.4 (334)	39.7 (274)
Investment	16.4 (193)	25.4 (175)
Injury^a^, % (*n*)	11.2 (132)	18.7 (129)

a In the last competition season with more than 2 months of rehabilitation.

There was a significant association between the Stages and Injury (χ^2^(2,*N* = 1864) = 102.94, *p* < 0.001, V = 0.24). In the Investment stage, the athletes were more likely to have sports injuries (27.4%, *n* = 101) than in the Sampling (6.3%, *n* = 56) and Specializing (16%, *n* = 97) stages.

### PISQ in the parents’ and athletes’ samples

The means, standard deviations, Hedges’ g and Cronbach’s αs that were obtained for the PISQ subscales are presented in Table 2. Both parental and athlete versions of the PISQ showed adequate internal consistency (Cronbach’s α = 0.61 to 0.86).

**Table 2:** Mean, standard deviation, and Cronbach’s α of the subscales of the PISQ.

	Cronbach α		Parents		Athletes		*t*	*p*	g
	Parents (*n* = 1,174)	Athletes (*n* = 690)	M	SD	M	SD			
DB	0.86	0.83	2.73	1.02	3.18	1.20	8.306	<0.001	0.41
PU	0.61^a^	0.81	4.67	0.60	4.37	0.75	−8.993	<0.001	0.45
AI	0.63^a^	0.66^a^	2.48	1.21	2.28	1.15	−3.430	0.001	0.17
PP	0.64	0.72	2.09	0.88	1.94	0.93	−3.519	<0.001	0.17

a The subscales contain two items.

DB, Directive Behavior; PP, Parental Pressure; AI, Active Involvement; PU, Praise and Understanding; g, Hedges’ g.

Athletes report significantly lower PU, AI and PP and higher DB than parents. Although significant differences are found between parents and athletes on all four forms of involvement, the difference in means between the DB and PU subscales can be interpreted as significant in practice, and we will therefore continue to focus on these two subscales.

### The effects of samples, stages and injury on parental involvement

#### Directive behavior

There was a significant main effect of Stages (*F*(2,1852) = 10.079, *p* < 0.001, η^2^_p_ = 0.01), indicating that greater DB was reported in the Sampling (M = 3.18, SE = 0.08) compared to the Investment (M = 2.75, SE = 0.06) and Specializing stages (M = 2.83, SE = 0.06). There was a non-significant main effect of Injury (*F*(2,1852) = 0.023, *p* = 0.878, η^2^_p_ = 0.00). There was a non-significant Stages × Injury 2-way interaction (F(2,1852) = 3.107, *p* = 0.045, η^2^_p_ = 0.00). The result showed a significant Samples × Injury × Stages 3-way interaction (*F*(6,1852) = 16.250, *p* < 0.001; η^2^_p_ = 0.05).

**Figure 1:**
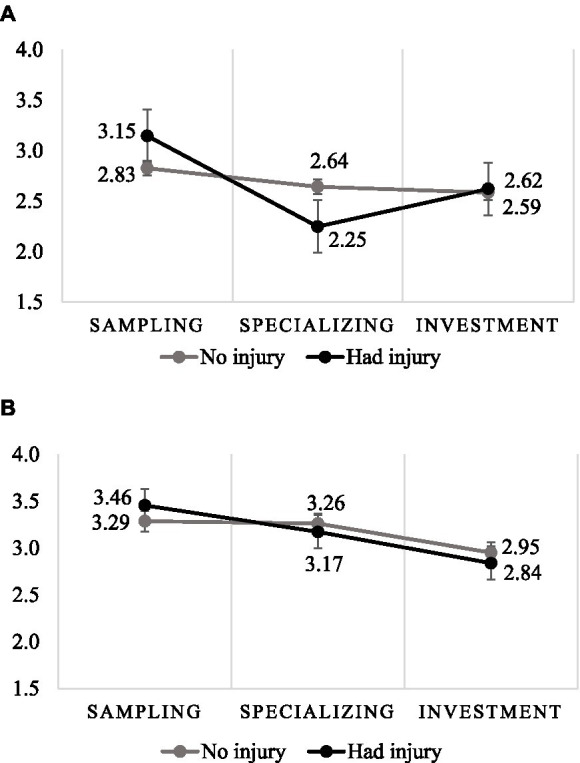
Directive behavior in stages by injury among parents **(A)** and athletes **(B)** (error bar: standard error).

The main effect of Stages is only seen in parents whose child has been injured. There is no difference in the sample of athletes, either by Injury or by Stages, Figure 1.

#### Praise and understanding

There was a significant main effect of Stages (F(2,1852) = 4.009, *p* = 0.028, η^2^_p_ = 0.00). PU was higher in the Sampling (M = 4.56, SE = 0.05) PU than in the Investment stage (M = 4.40, SE = 0.04). The Specializing stage (M = 4.49, SE = 0.04) showed a non-significant difference from the Investment and Sampling stages.

The Injury main effect was significant (*F*(1,1852) = 3.597, p = 0.028, η^2^_p_ = 0.00), as those reporting a sports injury (M = 4.44, SE = 0.04) perceived a lower level of PU than who had not been injured (M = 4.53, SE = 0.02).

**Figure 2:**
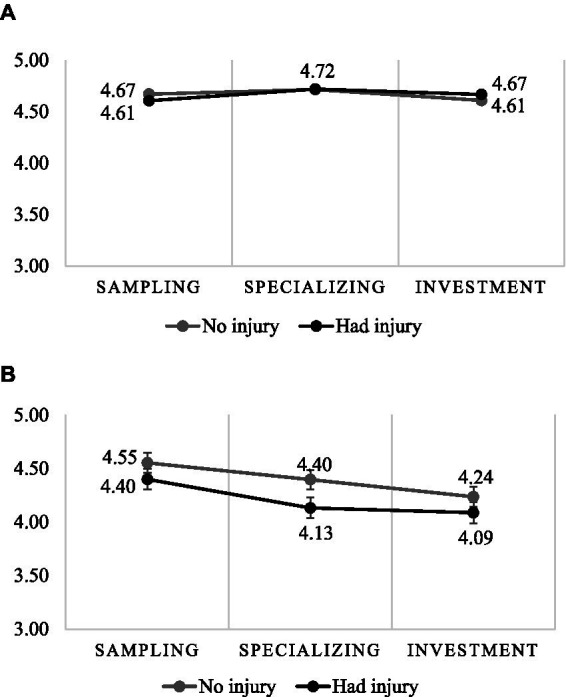
Praise and understanding in stages by injury among parents **(A)** and athletes **(B)** (error bar: standard error).

There was a non-significant Stages × Injury 2-way interaction (F(2,1852) = 0.335, *p* = 0.715, η^2^_p_ = 0.00). The result showed a significant Stages × Injury × Samples 3-way interaction (F(6,1852) = 16.011, *p* < 0.001, η^2^_p_ = 0.05). In the sample of injured athletes, PU was lower in the Specializing stage. There is no difference in the sample of parents, either in terms of Injury or Stages (Figure 2).

## Discussion

Our research was conducted among parents and youth handball players and aimed to investigate the factors that predicted changes in the type and extent of parental involvement. The parental involvement study explored both agents’ (athletes’ and parents’) perspectives concurrently on parental involvement. It was found that the interaction models of both subscales (namely, DB and PU) were significant. The results showed that the degree of parental involvement is influenced by the sample (athlete or parent), sport participation stage, and previous sports injuries.

Consistent with previous studies (Giannitsopoulou et al., 2010; Marsh et al., 2015; Goodman and James, 2017), we found a difference between the two perspectives, athletes perceiving PU lower and DB higher than parents. Findings suggest that athletes perceive their parents as exerting higher levels of control and lower levels of parental support compared to the parents’ perceptions. Studies have demonstrated that in certain sports, such as gymnastics and football, young individuals would prefer to experience less directive, controlling parental behavior (Giannitsopoulou et al., 2010; Mastrorilli and Greco, 2020). In other sports, such as swimming (Rodis, 2013), youth athletes are satisfied with the current level or would appreciate more control (Marsh et al., 2015). Researchers hypothesize that this phenomenon may result not only from variations between sports but also from age-related traits.

Our study identified differences between the two perspectives when comparing sport participation stage. Among the parents, comparing sport participation stage revealed that only those who had a child athlete with an injury displayed decreased DB. In contrast, the levels of DB for parents of non-injured athletes did not change, and the level of supportive parental behavior remained constant across the age groups. In contrast, both PU and DB reported by athletes in different sport participation stage decreased, with higher levels in the sampling stage than in the specializing stage.

The perceptions of parental involvement in the athlete sample exhibit different dynamics than the parents’ self-perceptions. Our findings partially support the claim that parental involvement diminishes as their child progresses in their career, as asserted by athlete development models such as DMSP (Côté and Hay, 2002) and HAC (Wylleman and Lavallee, 2004). This process may be underpinned by a natural phenomenon on the athlete’s side, whereby feedback from the environment, peers and coaches, becomes increasingly crucial as the young athlete ages. During the young athletes’ early years, parents act as role models by exemplifying physical activity, creating conditions for sports play, and fostering the necessary environment to develop the athletes’ intrinsic motivation. Later on, coaches and sports partners may take over part or all of this guiding role. Young athletes may also perceive parental involvement differently as they grow older.

Parents’ roles and responsibilities remain both constant and multifaceted over time (Gould et al., 2008; Knight and Holt, 2012, 2014; Harwood and Knight, 2016). While some studies emphasize the importance of parental involvement during the early stages of a child’s athletic pursuits (Sukys et al., 2014; Lawler et al., 2022), much of the research has focused on the athlete’s viewpoint rather than the parents’. Our study aimed to provide insights into the experiences of parents and athletes in youth sports.

Our results demonstrate that injury is associated with a decreased level of perceived PU across age groups in both the parent and athlete samples. In line with the Multilevel Model of Sport Injury (MMSI, Wadey et al., 2018), based on Bronfenbrenner’s bioecological model, five distinct levels: Interpersonal, Intrapersonal, Institutional, Cultural and Policy. The Intrapersonal level reflects the characteristics of the individual such as age, gender, attitudes, etc. The Interpersonal level focuses on formal and informal networks and support systems such as coaches, parents, teammates, etc., and the interactions between them. Based on MMSI’s Intrapersonal characteristics, the risk of injury is highest in team sports during youth (Schwebel and Brezausek, 2014). In addition, playing handball is associated with a high risk of injury (Moller et al., 2012). Padaki et al. (2017) found that athletes who train more than 9 h per week are more likely to be injured than those who train less. Training volume may determine not only the risk of sports injury but also the parent’s behavior. Studies indicate that the higher the level of exercise a child engages in, the more likely it is to have a direct influence on a parent’s involvement in their child’s sporting life. However, our findings suggestthat parents’ DB decreases when their child suffers a severe sports injury that required a rehabilitation period of at least 2 months. Few studies have addressed the impact of sports injury on the athlete-parent dyad dynamic, as reflected in the MMSI’s Interpersonal level. Oosterhoff et al. (2018) investigated the impact of sports-related orthopaedic injuries on 54 adolescent athlete-parent dyads. Based on their results, parents are not likely to have an overly negative attitude towards their child’s subjective emotional response to the injury. In contrast, according to another study, parental distress may be a predictor of the likelihood of injury in young athletes (Schwebel et al., 2011).

Our findings also revealed discrepancies between the athletes’ and parents’ perceptives. Athletes reported a decline in PU, while parents reported a decline in DB in the case of injury. This result may provide a valuable research direction. These behaviors (DB and PU) might be part of the same construct/parenting style. Baumrind (1971) defined authoritative parenting as having a balance of control and emotional warmth, and linked it to positive outcomes, such as the young athlete’s healthy perfectionism (Sapieja et al., 2011) and motivation (Juntumaa et al., 2005). Parents might experience stress due to their child’s injury, leading to changes in their behavior and involvement with their child’s activities. The athlete may experience this change in behavior differently than the parent does. Our findings could offer a new way to explore parental involvement and highlight the dynamics and characteristics of parent-athlete interaction. Our results also emphasize that injuries might induce changes in the behavior of both athletes and parents. It is important to provide practices for parents to help find an alternative way to support their children after sports injury. Further longitudinal and qualitative studies are required to analyze changes in perceived supportive parental behavior in different developmental phases. In addition, parents need special information and support from coaches and the medical team to avoid decreasing parental involvement from the specializing stage.

### Limitations

As our study is cross-sectional, it is crucial to emphasize the research’s limitations concerning the comparison of stages in the athletic life cycle. To reinforce our results, a longitudinal study is necessary to monitor variations in parental involvement over time. Parenting is, of course, not confined to parents’ involvement in their children’s sports career, while the intensity of parental involvement itself may be influenced by various factors (e.g., results and longevity of the sporting season, child’s overtraining or burnout). For sport-related injuries, the exact type was not specified. However, the absence of 2 months of training suggests a significant injury that could potentially influence drop-out. In this research, the causes (e.g., overuse, overtraining) and consequences (e.g., burnout, dropout) of the injuries were not examined, it would be a suitable next step in a follow-up study. It is noteworthy that we did not match parents with their children in our research. In subsequent studies, it is essential to link the views of athletes with those of their parents to better comprehend the gap between perspectives. Our study did not address the impact of parents’ previous experience on their involvement, despite over 55% of the participating parents having a history of athletic participation, a factor that may have influenced their participation traits (Holt et al., 2008). More research is necessary to explore the potential effect of parents’ previous experience, in particular, whether they participated in the same sport as their child. The over-representation of women/mothers in our survey is a further limitation which decreases the generalization of the results for parents. Finally, data were collected between June 2022 and July 2022, and this time was one of the first full seasons back after the global COVID-19 pandemic which can influence the results; at the same time, from 7 March 2022, all restrictions have been abolished; thus, the state of affairs before the coronavirus pandemic has been restored.

## Conclusion

This study focuses on parental involvement as perceived by both parents and athlete children through a quantitative research lens. The results of our study indicate that both a parent’s identity and the athlete’s age group and injury status can influence the two forms of parental involvement. Parents’ self-perceived level of involvement can differ from the involvement perceived by an athlete child. Although athletes’ perceptions of parental involvement decline, parents’ self-perceptions of involvement remain unchanged when comparing age groups. If athletes are injured, (perceived) parental behavior may change during the specializing stage. Athletes perceive a decrease in Praise and Understanding, whereas for parents, this results in a decrease in Directive Behavior. Our findings suggest that these two behaviors could be part of the same parenting style, which requires further investigation. These findings have important implications for parental psychoeducation as well as for the training of sports professionals such as coaches, sports medicine physicians, and rehabilitation experts.

## Data availability statement

The raw data supporting the conclusions of this article will be made available by the authors, without undue reservation.

## Ethics statement

The studies involving humans were approved by Research Ethics Committee of the ELTE PPK; with license number 2022/321. The studies were conducted in accordance with the local legislation and institutional requirements. Written informed consent for participation in this study was provided by the participants’ legal guardians/next of kin.

## Author contributions

KrK: Conceptualization, Formal analysis, Investigation, Methodology, Writing – original draft, Writing – review & editing. JT: Formal analysis, Supervision, Visualization, Writing – original draft, Writing – review & editing. IJ: Data curation, Writing – review & editing. KaK: Conceptualization, Data curation, Writing – review & editing.

## Footnotes

^1^ http://www.statskingdom.com
